# Odevixibat treatment reverses severe phenotype of PFIC in a young adult: A real-life experience beyond the genetic diagnosis

**DOI:** 10.1016/j.jhepr.2025.101486

**Published:** 2025-06-10

**Authors:** Claudia Mandato, Mario Masarone, Mariano Festa, Pietro Torre, Marcello Persico

**Affiliations:** 1 Pediatric Hepatology, Department of Medicine, Surgery and Dentistry “Scuola Medica Salernitana”, Pediatrics Section, University of Salerno, Baronissi Salerno, Italy; 2 Internal Medicine, Department of Medicine, Surgery and Dentistry “Scuola Medica Salernitana”, Pediatrics Section, University of Salerno, Baronissi Salerno, Italy

To the Editor:

Progressive familial intrahepatic cholestasis (PFIC) encompasses a group of rare disorders that typically manifest in childhood with severe cholestasis. The spectrum of PFIC has expanded significantly in recent years. In adults, they exhibit considerable variability, with gene mutations being associated with cholestasis of pregnancy, low-phospholipid cholelithiasis, severe recurrent cholestasis, and recurrent drug-induced liver injury.^1^ Recently, the European Association for the Study of the Liver published guidelines on the diagnosis and treatment of cholestatic diseases in children and adults.^2^ The ileal bile acid transporter inhibitors (IBATi) odevixibat and maralixibat have been shown to effectively alleviate pruritus and reduce serum bile acid levels in children with PFIC, demonstrating high tolerability,^3^ including in real-life experiences.^4^

Severe cholestasis can be further complicated by cholemic nephropathy (CN), or bile cast nephropathy, a rare but severe condition. Its pathophysiological mechanisms remain poorly understood. It may be caused by severe hyperbilirubinemia and bile acid accumulation, with tubular toxicity, bile cast formation, oxidative stress, and renal hypoperfusion.^5^^,^^6^ Current treatment approaches typically include dialysis and plasmapheresis.^7^ Renal sodium-dependent bile acid transporter blockade prevents CN in animal models of obstructive cholestasis.^8^

A recent algorithm suggests initiating IBATi therapy in patients with clinical and biochemical features indicative of PFIC, even before genetic testing results are available, particularly in critically ill patients.^9^

We report the case of a 27-year-old female presenting with severe hyperbilirubinemia, CN, and liver failure, successfully managed with odevixibat, who was later diagnosed with PFIC.

The patient was admitted for severe jaundice and intractable pruritus. Her clinical history included transient, asymptomatic hypertransaminasemia of unknown origin at age 15 (no clinical documentation available). Family history was unremarkable, and no hepatopathy or cholestasis of pregnancy were reported. Five months before admission, she started taking levonorgestrel/ethinylestradiol for dysmenorrhea. After 2 months, she developed pruritus, leading her to discontinuation. Approximately 3 weeks later, jaundice appeared.

On admission, she was jaundiced and presented scratch injuries. Laboratory tests revealed conjugated hyperbilirubinemia (total bilirubin 6.4 mg/dl, direct bilirubin 4.8 mg/dl), hypertransaminasemia (aspartate aminotransferase of 1.5x the upper limit of normal [ULN], alanine aminotransferase of 2x ULN, alkaline phosphatase of 2.5x ULN, and mildly-elevated gamma-glutamyltransferase [GGT] of 1.2x ULN). Viral and autoimmune causes were excluded. Ultrasound revealed gallbladder stones. Endoscopic retrograde cholangiopancreatography and laparoscopic cholecystectomy were performed, with a diagnosis of biliary cholelithiasis complicated by drug-induced liver injury (DILI).

Despite treatment with cholestyramine and ursodeoxycholic acid, her hyperbilirubinemia worsened (total bilirubin 12.2 mg/dl, direct bilirubin 9.2 mg/dl), while magnetic resonance imaging excluded hepatic or biliary tract lesions. Liver biopsy showed mild inflammatory infiltrates, fibrosis, focal steatosis, cholestasis, and focal bile duct degeneration (Metavir score F1). BSEP immunostaining was performed according to El Guindi *et al.* and was negative (Fig. 1B).^10^ Her condition further deteriorated with severe mixed hyperbilirubinemia (up to 40.03 mg/dl), elevated serum bile acids (262 μmol/L), rising creatinine levels (1.2 mg/dl). Due to prolonged exposure to high levels of bile acids and bilirubin and the worsening of renal failure we suspected bile cast nephropathy. She underwent four sessions of plasmapheresis, which provided temporary relief but were complicated by a subclavian artery rupture during the final procedure. Subsequently, her liver and renal function worsened (international normalized ratio 2.04, creatinine 1.69 mg/dl), and she was listed for liver transplantation with a MELD 3.0 score of 25.

**Fig. 1:**
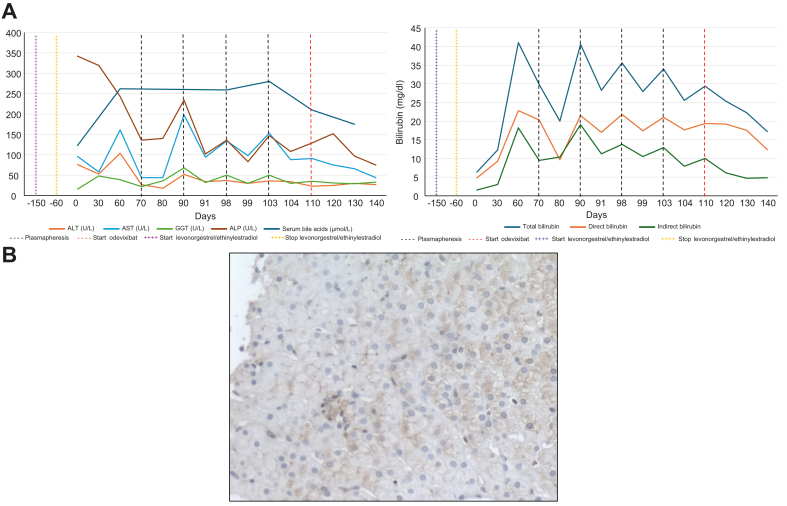
Biochemical trends of the patient over time and BSEP immunostaining. (A) Both panels demonstrate the impact of therapeutic interventions on the patient's biochemical markers. The top panel illustrates the levels of total bilirubin (blue line), direct bilirubin (orange line), and indirect bilirubin (green line) measured in mg/dl over the course of 140 days. Key events are marked with vertical dashed black lines, indicating sessions of plasmapheresis, and a vertical red dashed line, denoting the initiation of odevixibat therapy. The lines with “+++” represent the initiation of therapy (purple) with levonorgestrel/ethinylestradiol and the discontinuation due to pruritus (yellow), respectively. Time 0 corresponds to hospital admission. The bottom panel shows the trends for other biochemical parameters: AST (blue line), ALT (orange line), GGT (green line), ALP (brown line), and serum bile acids (dark blue line). The same events, plasmapheresis sessions (black dashed lines) and initiation of odevixibat therapy (red dashed line), levonorgestrel/ethinylestradiol (+++ purple and yellow line) are indicated. (B) BSEP immunostaining was performed according to El-Guindi *et al.* and was negative. Only scanty and very rare positive spots were observed in some hepatocytes. ALP, alkaline phosphatase; ALT, alanine aminotransferase; AST, aspartate aminotransferase; GGT, gamma-glutamyltransferase.

Given the elevated bile acids with a near-normal GGT and suspicion of PFIC, genetic testing was initiated, and treatment with odevixibat (2,400 μg/day) was started. Within weeks, odevixibat markedly improved her hyperbilirubinemia, pruritus, and liver and renal function, allowing her to be removed from the transplantation list. Compared to plasmapheresis, odevixibat provided sustained reductions in bilirubin and bile acids with a superior safety profile (Fig. 1A).

Liver stiffness measurement – performed after 1 month of odevixibat therapy, at the time of bilirubin decrease – was 7.1 kPa, confirming the Metavir biopsy score of F1 at the onset of the overt clinical manifestation.

Genetic analysis by next-generation sequencing (including 76 cholestasis-related genes) revealed a heterozygous *ABCB11* variant (maternal c.3669G) previously reported in 0.0055% of alleles in European individuals (non-Finnish) with cholestasis,^11^^,^^12^ and a heterozygous *ABCB11* variant (paternal c.221_222delinsAC) resulting in an in-frame deletion-insertion (p.Ile74Asn).This rare variant has a frequency of 1:249,000 alleles in the gnomAD public population database (reported as two separate variants: https://gnomad.broadinstitute.org/variant/2169869950AT and https://gnomad.broadinstitute.org/variant/2 169869949 TG). p.V444A polymorphism, often in trans in DILI-induced BSEP-deficiency, was not reported. The patient was also heterozygous for a *SERPINA1* mutation (c.221T).

This case underscores the pivotal role of early odevixibat in treating severe hyperbilirubinemia complicated by CN. It demonstrates the potential of IBATi as a therapeutic option for cholestatic DILI and CN in selected cases, if the phenotype suggests PFIC (low GGT cholestasis). Furthermore, it highlights the importance of initiating treatment on clinical suspicion, even before confirming genetic testing results, to halt progression towards end-stage liver disease. This case also expands the understanding of PFIC as a condition that can manifest in adulthood, often triggered by factors such as estrogen-progestin-induced liver injury. Due to the severity of the phenotype and the incomplete normalization of liver function tests, we did not consider discontinuing treatment after the acute episode; however, this remains a topic of debate.

In conclusion, early odevixibat treatment in patients with PFIC, with acute severe cholestasis and CN, can significantly improve cholestasis and prevent the need for liver transplantation. This case emphasizes the necessity of considering genetic testing and novel therapies in the management of cholestatic liver diseases when common etiologies are excluded.

## Financial support

This study didn’t receive any funding on regards of the conceptualization, drafting and revision. IPSEN agreed to pay the article processing charges by transferring the corresponding funds to University of Salerno.

## Authors' contributions

CM, MM, MP: conceptualization, drafting and revision. MF, PT: data collection and analysis, drafting and revision.

## Data availability statement

Data will be made available upon reasonable request to the corresponding author.

## Conflict of interest

The authors declare no specific conflicts regarding this report.

Please refer to the accompanying ICMJE disclosure forms for further details.
